# Genetic diversity structure of western-type carrots

**DOI:** 10.1186/s12870-021-02980-0

**Published:** 2021-04-26

**Authors:** Katarzyna Stelmach, Alicja Macko-Podgórni, Charlotte Allender, Dariusz Grzebelus

**Affiliations:** 1 Department of Plant Biology and Biotechnology, University of Agriculture in Krakow, Al. 29 Listopada 54, 31-425 Kraków, Poland; 2 School of Life Sciences, University of Warwick, Warwick, UK

**Keywords:** *Daucus carota*, Genetic diversity, Population structure, Market classes, Root shape, SNP, *DcSto*, DAPC

## Abstract

**Background:**

Carrot is a crop with a wide range of phenotypic and molecular diversity. Within cultivated carrots, the western gene pool comprises types characterized by different storage root morphology. First western carrot cultivars originated from broad-based populations. It was followed by intercrosses among plants representing early open-pollinated cultivars, combined with mass phenotypic selection for traits of interest. Selective breeding improved root uniformity and led to the development of a range of cultivars differing in root shape and size. Based on the root shape and the market use of cultivars, a dozen of market types have been distinguished. Despite their apparent phenotypic variability, several studies have suggested that western cultivated carrot germplasm was genetically non-structured.

**Results:**

Ninety-three *DcS*-ILP markers and 2354 SNP markers were used to evaluate the structure of genetic diversity in the collection of 78 western type open-pollinated carrot cultivars, each represented by five plants. The mean percentage of polymorphic loci segregating within a cultivar varied from 31.18 to 89.25% for *DcS*-ILP markers and from 45.11 to 91.29% for SNP markers, revealing high levels of intra-cultivar heterogeneity, in contrast to its apparent phenotypic stability. Average inbreeding coefficient for all cultivars was negative for both *DcS*-ILP and SNP, whereas the overall genetic differentiation across all market classes, as measured by F_ST_, was comparable for both marker systems. For *DcS*-ILPs 90–92% of total genetic variation could be attributed to the differences within the inferred clusters, whereas for SNPs the values ranged between 91 to 93%. Discriminant Analysis of Principal Components enabled the separation of eight groups cultivars depending mostly on their market type affiliation. Three groups of cultivars, i.e. Amsterdam, Chantenay and Imperator, were characterized by high homogeneity regardless of the marker system used for genotyping.

**Conclusions:**

Both marker systems used in the study enabled detection of substantial variation among carrot plants of different market types, therefore can be used in germplasm characterization and analysis of genome relationships. The presented results likely reveal the actual genetic diversity structure within the western carrot gene pool and point at possible discrepancies within the cultivars’ passport data.

**Supplementary Information:**

The online version contains supplementary material available at 10.1186/s12870-021-02980-0.

## Background

Cultivated carrot is a biennial root vegetable grown around the world in temperate and subtropical regions. It is an outcrossing diploid with a relatively small genome of ca. 473 Mb organized into 2n = 18 chromosomes [1]. Carrot is important nutritionally, placed among the most significant sources of β-carotene in the human diet. It is among the top ten vegetables in terms of global production [2]. Carrot is indigenous to Europe, Asia and North America, with Central Asia identified as the place of origin of cultivated carrots [3]. Carrot was most probably domesticated as a root crop around 1100 years ago in Central Asia. Early domesticated carrots were purple and yellow [4]. The majority of cultivated types that formed the basis of modern commercial cultivars were developed in Asia Minor (Turkey) and temperate regions of Europe. Thus, the above-mentioned regions are considered as the secondary centre of origin for carrot [5]. To date, several molecular approaches, such as isoenzymes, ALFPs, RFLPs and SNPs have been used to examine genetic relationships within *D. carota* [3, 6–8]. Population structure comprising four major groups has been commonly observed within *D. carota* species [3, 9–11]. European wild *D. carota* group is characterized by a high level of diversity and includes several *D. carota* subspecies; Asian wild group is less complex and comprises mostly *D. carota* subsp. *carota*. Western cultivated carrots form a numerous group characterized by high level of diversity in terms of storage root characteristics but are generally orange. Eastern cultivated carrots have more uniform root characteristics but display more variability in terms of colour, as they usually have yellow or purple roots. Further geographic structure was identified by Arbizu et al. [12], leading to the separation of an additional two groups: wild carrots of the Iberian Peninsula and Morocco (1) and landraces of the Balkan Peninsula, Middle East and North Africa (excluding Morocco) (2). Investigation of nearly 120 accessions representing Chinese cultivars and western cultivated carrot carried out by Ma et al. [13] showed clear separation of both gene pools and suggested independent processes of carotenoid-based root pigmentation in the history of eastern cultivars development.

Carrot is a crop with a wide range of phenotypic and genotyping variation that might be of use to breeders. Since the seventeenth century, a lot of breeding efforts have been focused on root traits such as shape, smoothness of the root surface or root integrity [14, 15]. First carrot cultivars originated from broad-based populations. It was followed by intercrosses among plants representing early open-pollinated (OP) varieties, combined with mass phenotypic selection for traits of interest. Discovery of cytoplasmic male sterility in the late 1940s led to a shift from OP to hybrid cultivars characterized by higher level of uniformity. Nonetheless, OP cultivars are still a valuable source of genetic diversity and represent a large portion of plant materials freely available to breeders worldwide through gene banks and public breeding programs [16].

**Fig. 1:**
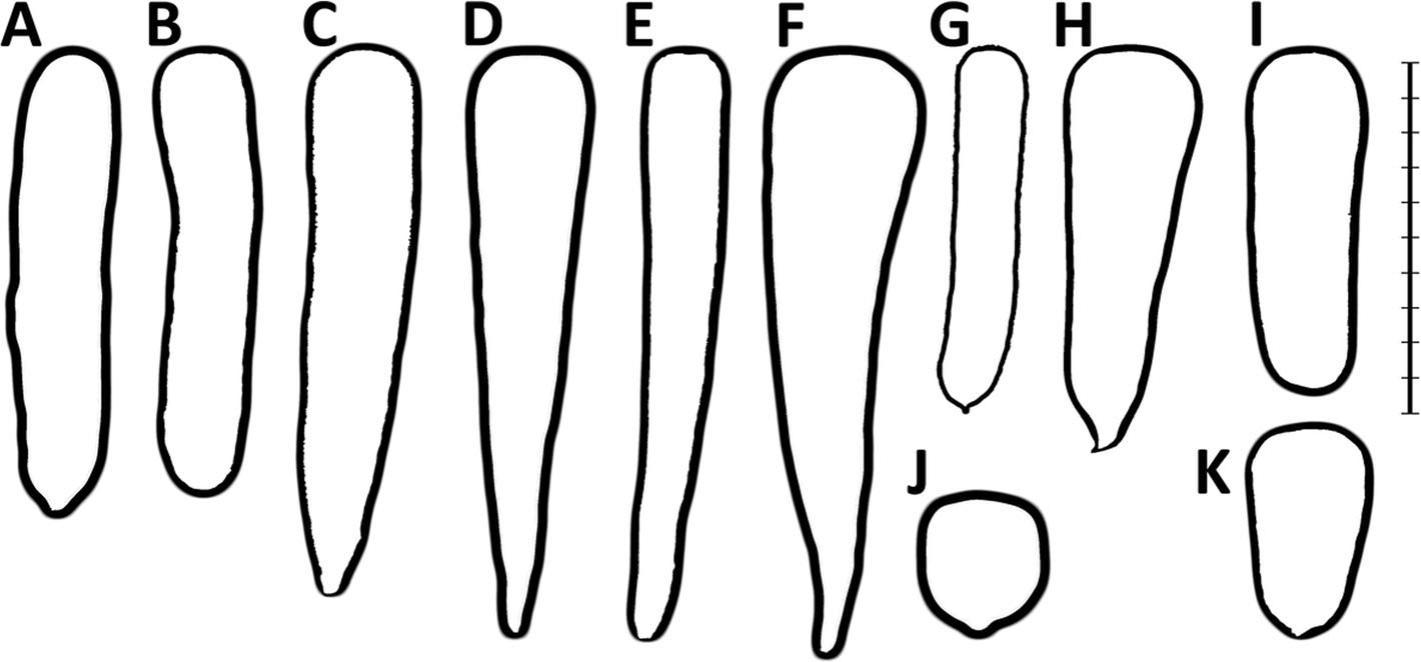
Typical shapes of carrot storage roots representing some of the most popular market types. A - Nantes, B - Berlikum, C - Autumn King (=Flakkee), D – Long Orange, E - Imperator, F – St. Valery, G - Amsterdam, H - Danvers, I - Chantenay, J – Paris Market, K - Guérande (=Oxheart). Bar length – 20 cm

Selective breeding improved root uniformity and led to the development of a range of cultivars differing in root shape and size. Based on the root shape and the market use of carrot cultivars, a dozen of market types (or varietal groups) have been distinguished [17] (Fig. 1). Some older market types were typically bred and developed in Europe (e.g. Long Orange, Amsterdam or Paris Market), while others were characteristic for the U.S. market (e.g. Imperator or Danvers). The work of Banga [14] has been the most comprehensive description of the main western cultivated carrot types available to date. He described key characteristics and use of modern types of western carotene carrot and discussed their connection with well-established original varieties. Despite apparent phenotypic variability observed among market classes, several studies have suggested that western cultivated carrot germplasm was genetically non-structured [3, 9, 10]. Later studies carried out by Stelmach et al. [18] provided the first molecular evidence for a possible root-type associated structure of genetic diversity in western cultivated carrot. They showed that *Daucus carota Stowaway (DcSto)* Miniature Inverted Repeat Transposable Element (MITE) based molecular markers (*DcS*-ILP) detected substantial variation among carrot plants of different origin and could be exploited in germplasm characterization and analysis of genome relationships. MITEs are non-autonomous DNA transposons requiring the presence of a related autonomous element to be a donor of a transposase inducing their transposition *in trans*. Global analysis of *DcSto* MITEs provided evidence for their recent mobility and identified a candidate autonomous element, *DcMar1*, as a possible source of transposase [19].

In the present study, we investigated a collection of plants from a range of OP western-type carrot cultivars producing roots of different shapes and representing several varietal groups. We aimed to detect possible genetic structure underlying apparent phenotypic differences among well-established market types. We exploited and compared two codominant molecular marker systems, *DcS*-ILPs and SNPs, as we assumed that the former system might be capable of revealing variability which arose more recently, resulting from the transpositional activity of *DcSto* MITEs.

## Results

### Genetic diversity revealed by DcS-ILP and SNP genotyping

A total of 93 *DcS*-ILP markers and 2354 SNP markers, distributed along the nine carrot chromosomes (Additional file 1: Figure S1), were used to evaluate genetic diversity in the collection of 78 western type carrot cultivars. For *DcS*-ILP genotyping the number of alleles N_a_ was 1.676 and the number of effective alleles N_e_ was 1.411, whereas for SNP genotyping the corresponding N_a_ and N_e_ values were 1.783 and 1.512, respectively. The observed heterozygosity H_O_ was higher for SNPs (0.323) than for *DcS*-ILPs (0.253), as well as the expected heterozygosity H_E_ (0.295 for SNPs and 0.239 for *DcS*-ILPs). H_E_ estimates of the cultivars ranged from 0.115 (LC1) to 0.323 (BE7) for *DcS*-ILPs and from 0.174 (LO1) to 0.350 (GU3) for SNPs. (Additional file 2: Table S1 and Additional file 3: Table S2). The mean percentage of polymorphic loci segregating within a cultivar varied from 31.18% (LC1) to 89.25% (BE7) for *DcS*-ILP markers and from 45.11% (LC1) to 91.29% (SV1) for SNP markers (Additional file 4: Table S3) revealing high levels of intra-cultivar heterogeneity, in contrast to their apparent phenotypic stability. Cultivars belonging to the Amsterdam market class were characterized by the lowest mean within-group H_O_ (0.255 and 0.316 for *DcS*-ILP and SNP, respectively), whereas cultivars of the Imperator market class were characterized by the highest mean within-group H_O_ (0.277 for *DcS*-ILPs and 0.329 for SNPs; Additional file 5: Table S4). The within-group genetic diversity (measured as H_E_) was generally lower than H_O_ and ranged between 0.204 (Chantenay) and 0.267 (Berlikum) for *DcS*-ILP and between 0.269 (Chantenay) and 0.336 (St. Valery) for SNPs. Average inbreeding coefficient F_IS_ for all cultivars was negative for both *DcS*-ILP (− 0.055) and SNP (− 0.097), again indicating high levels of intra-cultivar heterogeneity. The overall genetic differentiation across all market classes, as measured by F_ST_, was comparable for both marker systems (0.294 for *DcS*-ILPs and 0.279 for SNPs; Table 1).

**Table 1:** F-statistics over all cultivars for all loci resulting from *DcS*-ILP and SNP genotyping

***DcS***-ILP				SNP			
	Fis	Fit	Fst		Fis	Fit	Fst
**Mean**	−0.055	0.252	0.294	**Mean**	−0.097	0.209	0.279
**SE**	0.014	0.013	0.007	**SE**	0.002	0.002	0.001

**Fig. 2:**
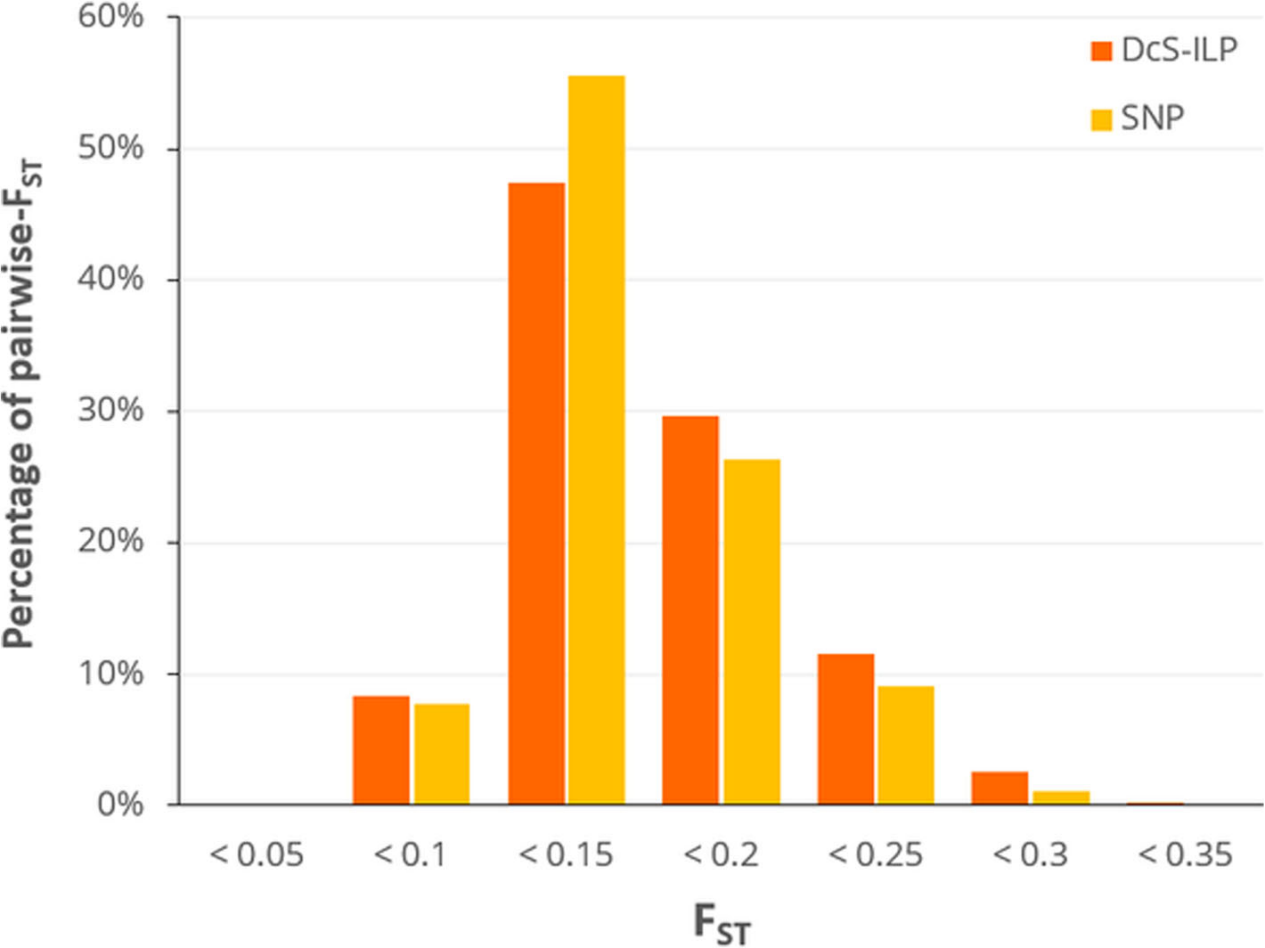
Comparison of the distribution of estimated pairwise F_ST_ between the 78 studied OP carrot cultivars

The F_ST_ analysis suggests a moderate level of differentiation between market classes. The strongest differentiation was observed between the cultivars belonging to the Amsterdam and St. Valery market classes (0.260 for *DcS*-ILP and 0.214 for SNPs; Additional file 5: Table S4). The within-group H_O_ values were higher than the pairwise F_ST_ values, indicating that within-cultivar genetic variability had greater contribution to overall diversity than between-cultivar variability. Pairwise F_ST_ estimates computed for pairs of cultivars were comparable for both marker systems and ranged from 0.046 (IM3 vs. IM5) to 0.332 (LC1 vs. AM1) for *DcS*-ILP markers, and from 0.052 (NA2 vs. NA3) to 0.323 (LC1 vs. AM1) for SNPs (Additional file 6: Table S5 and Additional file 7: Table S6). The highest percent of pairwise-F_ST_ ranged between 0.1 and 0.15 (47.5% for DcS-ILP and 55.6% for SNPs; Fig. 2). AMOVA of both *DcS*-ILP and SNP genotyping data showed there was more genetic variation observed within the studied cultivars (71% for *DcS*-ILP and 68% for SNPs) than among them (29% for *DcS*-ILP and 32% for SNPs), further underlying the presence of significant amounts of heterogeneity within carrot OP cultivars.

### Assessment of genetic structure using a model-based approach

**Fig. 3:**
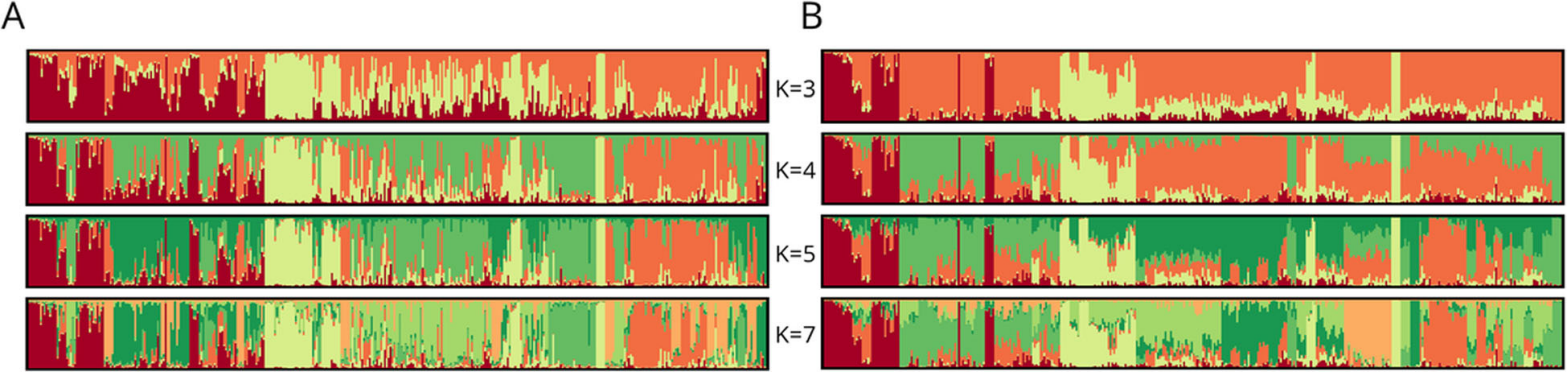
Estimated genetic structure of the 390 carrot plants representing 78 cultivars. A – genetic structure inferred using 93 *DcS*-ILP markers for K = 3–5 and K = 7; B – genetic structure inferred using 2354 SNP markers for K = 3–5 and K = 7. Each plant is represented by vertical line divided into colored segments representing the membership fractions in the K clusters

The possible genetic structure within western cultivated carrots was inferred without any prior classification. The entire collection of 390 carrot plants was analyzed using an admixture model-based clustering (Fig. 3). ΔK values (Additional file 8: Table S7) suggested that the most likely number of clusters for *DcS*-ILP genotyping was three, four or seven, with K = 4 being the most probable (ΔK = 61.498), while for SNP genotyping it was three, four and five, with K = 3 being the most probable (ΔK = 133.541). Therefore, a more detailed assessment and comparison of the genetic structure was conducted for K ranging from three to five, and for K = 7.

**Fig. 4:**
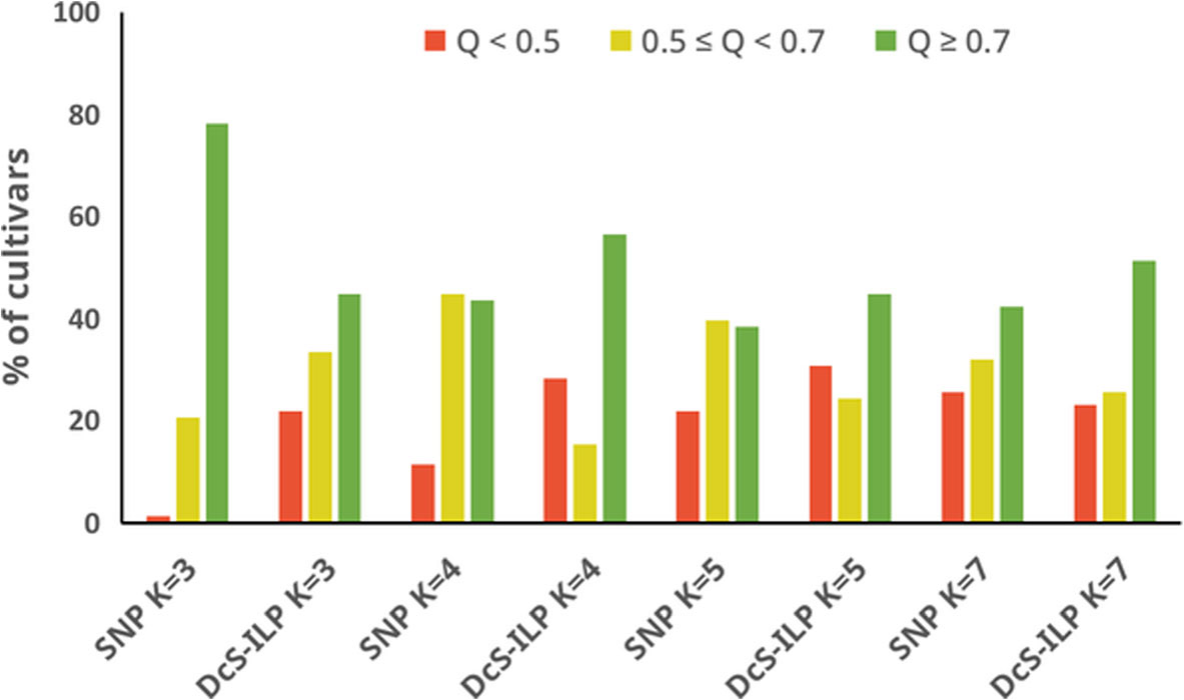
Percentage of cultivars assigned to the assumed clusters. Assignment is based on different thresholds (Q ≥ 0.5 and Q ≥ 0.7) of the membership coefficients using SNP and *DcS*-ILP markers for the most probable numbers of K: K = 3–5 and K = 7. Q < 0.5 indicates a high level of admixture within a cultivar

In general, the genetic structure inferred from the SNP data was characterized by a greater number of cultivars assigned to the assumed clusters (member coefficient values (Q) ≥ 0.5) as compared to *DcS*-ILP data. The percentage of populations assigned was as followed: 98.7% for K = 3, 88.5% for K = 4, 78.2% for K = 5 and 74.4% for K = 7. For *DcS*-ILP data the percentage was generally 10 to 20 points lower and varied from 69.2% for K = 5 to 78.2% for K = 3. However, the number of cultivars attributed to clusters with high confidence (> 0.7) was higher for the *DcS*-ILP data set when K was larger than 3, as compared to the SNP data set (Table 2**,** Fig. 4).

**Table 2:** The number of cultivars assigned to clusters (K) based on the value of membership coefficient (Q)

	SNP			***DcSto***		
	Q ≥ 0.7	0.7 > Q ≥ 0.5	Q < 0.5	Q ≥ 0.7	0.7 > Q ≥ 0.5	Q < 0.5
	number of cultivars					
K = 3	61	16	1	35	26	17
K = 4	34	35	9	44	12	22
K = 5	30	31	17	35	19	24
K = 7	33	25	20	40	20	18

Legend: Q ≥ 0.7 indicates low level of admixture; 0.7 > Q ≥ 0.5 indicates moderate level of admixture, whereas Q < 0.5 indicates high level of admixture. Cultivars with Q < 0.5 were not assigned to any of the inferred clusters

### SNP markers

**Fig. 5:**
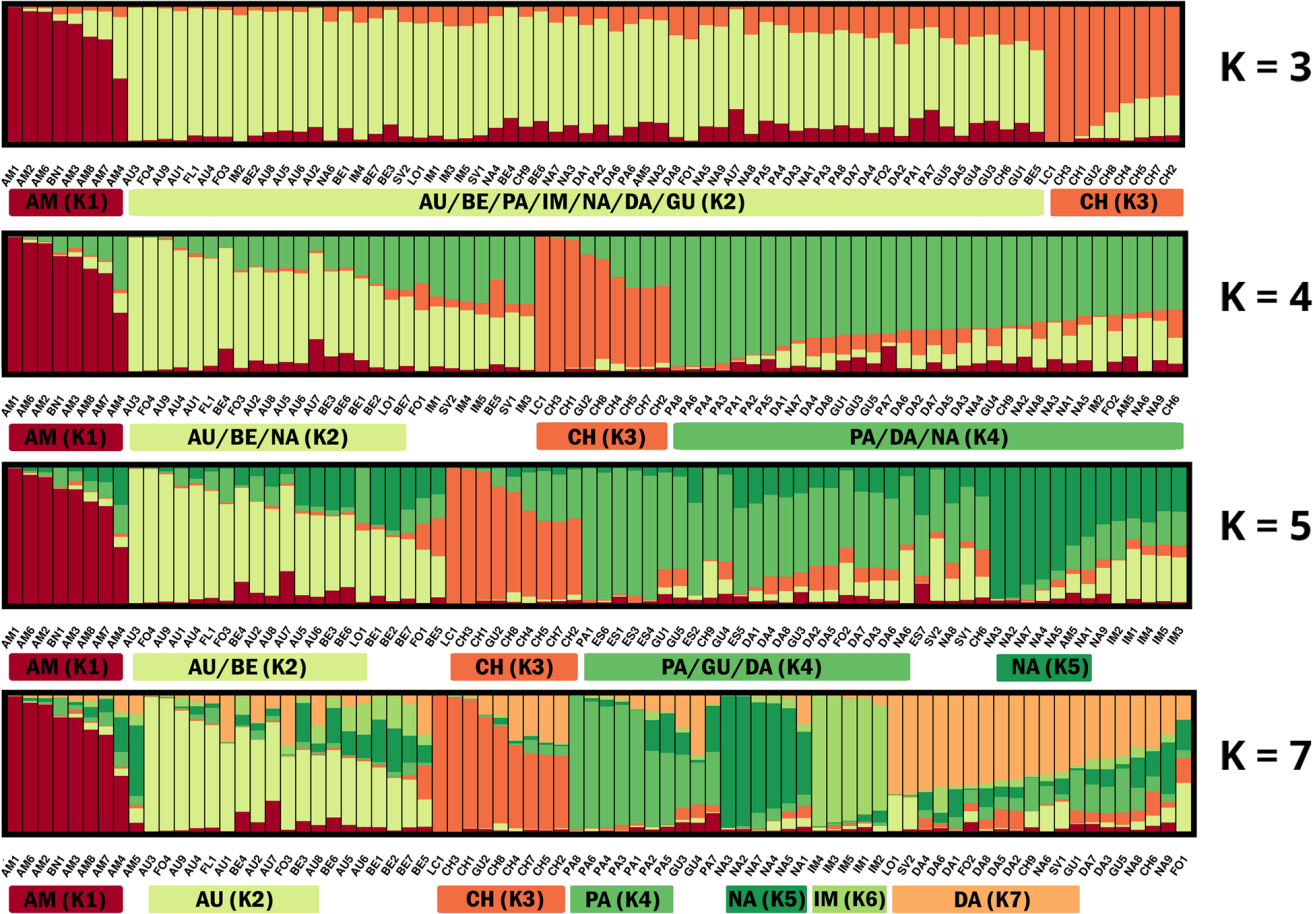
Estimated genetic structure of 78 carrot cultivars based on SNPs, inferred for K = 3–5 and K = 7. Two-letter abbreviations are used for indication of carrot market types: AM – Amsterdam, AU – Autumn King (Flakkee), BE – Berlikum, PA – Paris Market, IM – Imperator, NA – Nantes, DA – Danvers, GU – Guerande (Oxheart), CH – Chantenay, FO – fodder carrot. Three-letter abbreviations are corresponding to the cultivar symbols listed in Additional file 11: Table S10

For K = 3, the most probable number of clusters, clear separation of populations representing the Amsterdam and Baby Nantes market types was observed (group K1, Fig. 5). This pattern was noticeable regardless of the increasing number of the assumed clusters (up to K = 7). The average value of Q within K1 was very high (0.89; Table 3B). Only one cultivar of Amsterdam type (AM4) was characterized by high level of admixture, whereas cultivar AM5 was consequently grouped with cultivars belonging to the Nantes market type. The other clearly separated cluster (K3), that virtually did not change despite of increasing number of K, consisted of eight cultivars belonging to the Chantenay type. The average Q value within this group was high as well (0.78). Group K2 consisted of populations representing diverse market types, e.g. Autumn King (AU), Berlikum (BE) and Imperator (IM) characterized by long, stump roots; or Paris Market (PA), Guerande (GU), Danvers (DA) and Nantes (NA) typically of shorter (short to medium), thicker conical roots. The average distances between individuals within the assumed clusters (measured by H_E_) were highest for K2 (0.40), whereas for the clusters more homogeneous with respect to the origin of cultivars - K1 and K3, they were 0.31 and 0.30, respectively (Table 4B).

**Table 3:** Mean values of membership coefficient (Q) within inferred groups (*K1-K7*) of carrot cultivars

**A**	**Group**						
**No. of clusters**	***K1***	***K2***	***K3***	***K4***	***K5***	***K6***	***K7***
K = 3	0.74	0.70	0.79	–	–	–	–
K = 4	0.85	0.75	0.87	0.74	–	–	–
K = 5	0.84	0.64	0.87	0.74	0.75	–	–
K = 7	0.83	0.73	0.82	0.76	0.67	0.80	0.77
**B**	**Group**						
**No. of clusters**	***K1***	***K2***	***K3***	***K4***	***K5***	***K6***	***K7***
K = 3	0.89	0.79	0.81	–	–	–	–
K = 4	0.87	0.72	0.77	0.72	–	–	–
K = 5	0.86	0.72	0.76	0.68	0.78	–	–
K = 7	0.86	0.70	0.74	0.80	0.84	0.88	0.64

Legend: A - membership coefficient values obtained from *DcS*-ILP genotyping; B - membership coefficient values obtained from SNP genotyping; columns *K1* to *K7* represent groups of carrot cultivars; rows K = 3 to K = 7 represent the most probable number of groups inferred in the course of STRUCTURE analysis

**Table 4:** Average distances between individuals in the inferred groups (*K1-K7*) of carrot cultivars

**A**	**Group**						
**No. of clusters**	***K1***	***K2***	***K3***	***K4***	***K5***	***K6***	***K7***
**K = 3**	0.32	0.34	0.25	–	–	–	–
**K = 4**	0.28	0.33	0.24	0.33	–	–	–
**K = 5**	0.27	0.34	0.23	0.33	0.31	–	–
**K = 7**	0.26	0.34	0.22	0.28	0.28	0.31	0.33
**B**	**Group**						
**No. of clusters**	***K1***	***K2***	***K3***	***K4***	***K5***	***K6***	***K7***
**K = 3**	0.31	0.40	0.30	–	–	–	–
**K = 4**	0.31	0.37	0.28	0.39	–	–	–
**K = 5**	0.31	0.38	0.28	0.38	0.34	–	–
**K = 7**	0.31	0.35	0.27	0.34	0.33	0.35	0.34

Legend: A – average genetic distance obtained from *DcS*-ILP genotyping; B - average genetic distance obtained from SNP genotyping; columns *K1* to *K7* represent groups of carrot cultivars; rows K = 3 to K = 7 represent the most probable number of groups inferred in the course of STRUCTURE analysis

Increasing the number of clusters to four resulted in the separation of K2 into two separate clusters: the new cluster K2 comprised of AU/BE/FO and cluster K4 comprised of PA/GU/DA/NA. When assuming five clusters, six cultivars belonging to the Nantes market type were separated from K4 creating cluster K5. When the number of clusters was increased to seven, five cultivars belonging to the Imperator type were clearly separated (K6) with Q values above 0.85. The seventh group (K7) consisted mostly of cultivars attributed to the Danvers and St. Valery market types. When K = 7, for four cultivars at least one the plants representing the cultivar was assigned to the different cluster than the majority, thus represented different gene pool. Within another eight cultivars at least one of the plants representing the cultivar was admixed (Q < 0.5) and therefore could not be assigned to any of the defined clusters.

### *DcS*-ILP markers

**Fig. 6:**
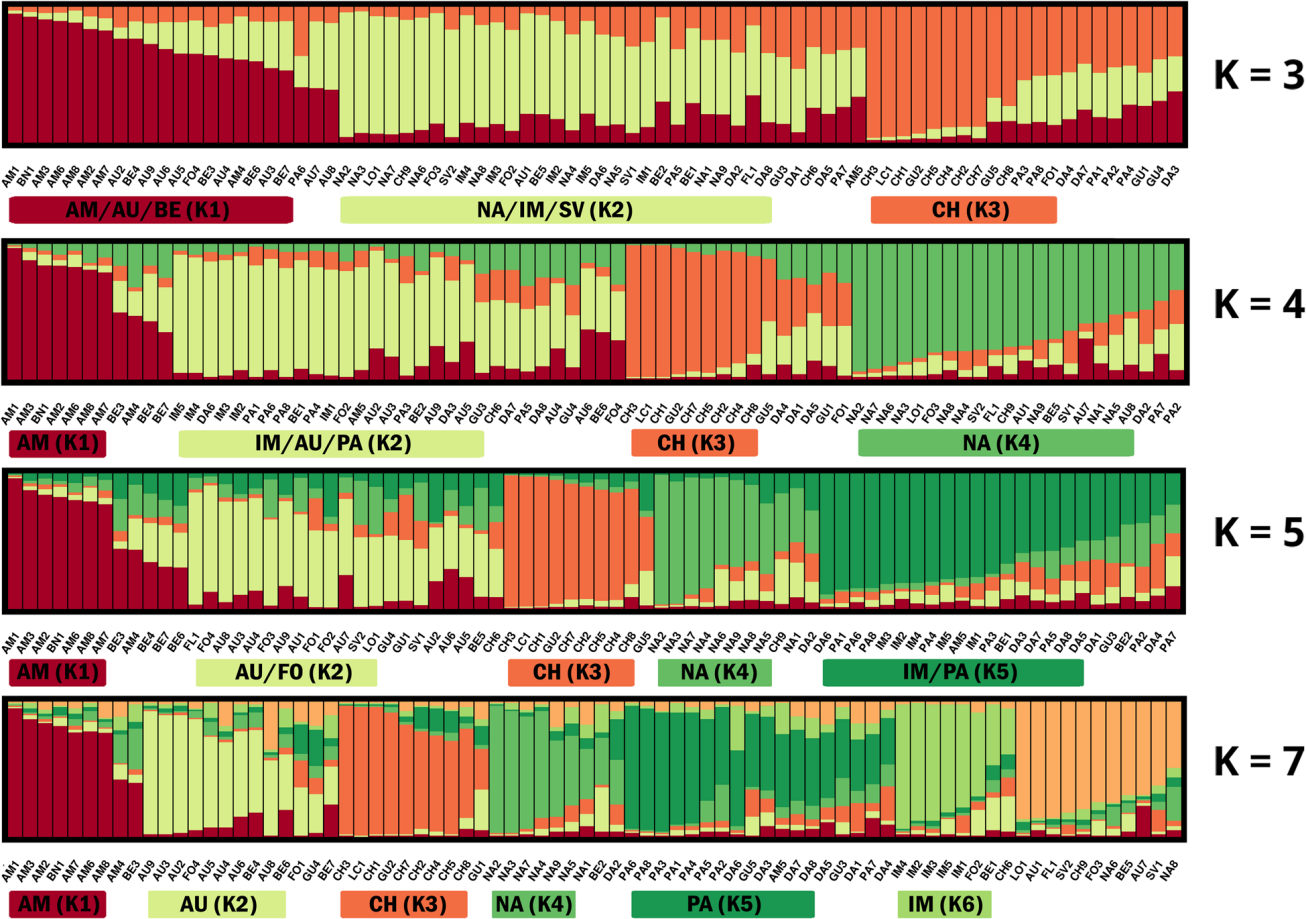
Estimated genetic structure of 78 carrot cultivars based on *DcS*-ILP markers, inferred for K = 3–5 and K = 7. Two-letter abbreviations are used for indication of carrot market types: AM – Amsterdam, AU – Autumn King (Flakkee), BE – Berlikum, PA – Paris Market, IM – Imperator, NA – Nantes, DA – Danvers, GU – Guerande (Oxheart), CH – Chantenay, FO – fodder carrot. Three-letter abbreviations are corresponding to cultivar symbols listed in Additional file 11: Table S10

When the presence of three clusters was assumed, two clusters (K1 and K2) grouped numerous populations of diverse origin such as Amsterdam, Berlikum, Autumn King in group K1 or Nantes, Imperator and St. Valery in group K2. Within K1 Amsterdam cultivars were characterized by high values of Q with mean of 0.85, whereas cultivars of other types were characterized by lower values of Q, not exceeding 0.77. The overall mean Q value within K1 was 0.74 (Table 3A). Eight out of ten analyzed cultivars of Chantenay market type were grouped with two cultivars of Guerande type and two cultivars of Paris Market type in one cluster K3 (Fig. 6). The mean Q for Chantenay cultivars within K3 was 0.89, whereas the overall mean Q for K3 was 0.79. When four clusters were assumed, the previous cluster K1 was reduced mainly to cultivars of the Amsterdam type, thus the mean Q value has increased to 0.85. Group K2 consistently comprised populations representing various root types such as Imperator, Autumn King and Paris Market with the mean Q value of 0.75. Group K3 was reduced to seven cultivars of Chantenay type and one belonging to Guerande market type and as such remained in spite of the increasing number of clusters (with mean Q = 0.87). For K = 4, newly separated group K4 consisted of 19 cultivars, nine of which belonged to the Nantes type (mean Q = 0.74). Increasing the assumed number of clusters to five resulted in separation of K5 (with mean Q = 0.75) from K2 group. K5 comprised of cultivars belonging to the Imperator and Paris Market types. For K = 5, K2 grouped seven cultivars belonging to the Autumn King market type, four populations of fodder carrot and single population of both Long Orange and St. Valery. The mean Q value for this cluster was 0.64. K4 was restricted exclusively to cultivars representing the Nantes type (mean Q = 0.74). When seven clusters were assumed, populations representing the Imperator type were grouped into K6 along with one population of fodder carrot (Q = 0.80). Group K7 was heterogeneous and comprised eleven cultivars of diverse market types. The mean Q value for that group was 0.77. For K = 7 17 cultivars were of mixed cluster assignment, whereas within another 22 cultivars at least one of the plants representing the cultivar was admixed (Q < 0.5) and therefore could not be assigned to any of the defined clusters. The average H_E_ between individuals within the assumed clusters were highest for K2 (0.34), whereas for the clusters more homogeneous with respect to the origin of cultivars - K1 and K3, they were of lower values (0.28 and 0.24, respectively; Table 4A).

For the most probable numbers of clusters, K = 3, K = 4 and K = 7 AMOVA analysis was carried out on both *DcS*-ILP and SNP genotyping data. In general, the majority of total genetic variation resulted from differences between cultivars assigned to the predefined clusters. For *DcS*-ILPs 90–92% of total genetic variation could be attributed to the differences within the clusters, whereas for SNPs the values ranged between 91 to 93%. For K = 3 and K = 4 the percentage of total variation resulting from differences between the assumed clusters was 7% for SNPs and 8% for *DcS*-ILPs. When the number of the predefined clusters was increased to seven, the percentage has risen to 9 and 10% for SNPs and *DcS*-ILPs, respectively.

### Discriminant analysis of principal components

**Fig. 7:**
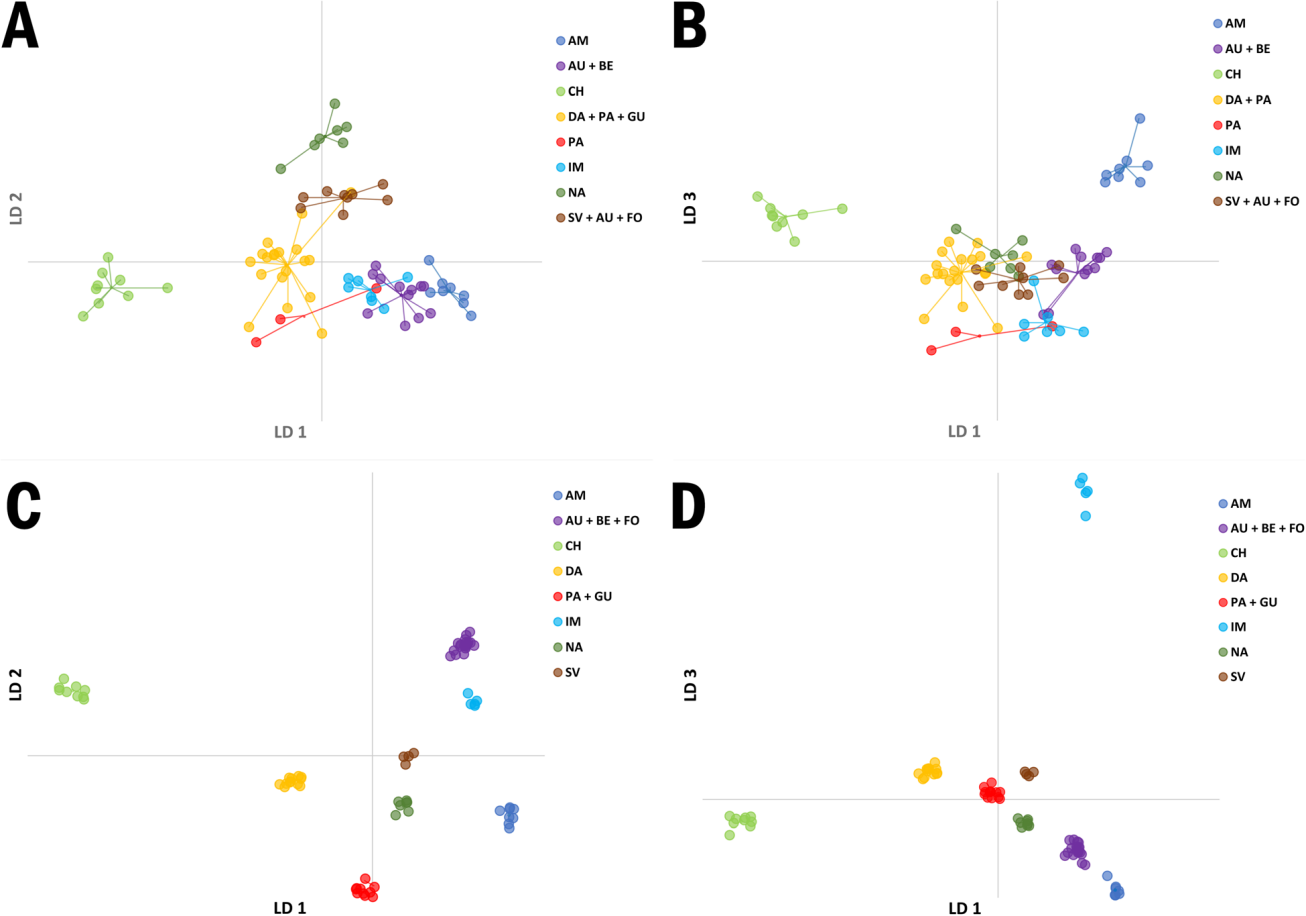
Discriminant analysis of principal components (DAPC) for 78 carrot cultivars. Analysis was carried out on *DcS*-ILP (**a, b**) and SNP (**c, d**) datasets. The axes represent Linear Discriminants (LD): 1st vs. 2nd (**A, C**) and 1st vs. 3rd (**b, d**). Each dot represents a cultivar. Two-letter abbreviations are used for indication of carrot market types prevailing in specific groups: AM – Amsterdam, AU – Autumn King (Flakkee), BE – Berlikum, PA – Paris Market, IM – Imperator, NA – Nantes, DA – Danvers, GU – Guerande (Oxheart), CH – Chantenay, FO – fodder carrot

The analysis was carried out to provide an alternative non-model de novo grouping of the studied cultivars. The optimal number of groups (K) was found to be eight for both data sets resulting in comparable results of the classification of cultivars (Fig. 7). 65.39% assignment accuracy was observed on the cultivar level, i.e. 51 cultivars were attributed to the same groups 1–8. 277 of 390 plants were assigned to the same groups, resulting in 71.03% assignment accuracy at the plant level. Generally, DAPC enabled the separation of cultivars depending on their market type affiliation. Three groups of cultivars were characterized by high homogeneity regardless of the marker system used for genotyping: Amsterdam (group 1), Chantenay (group 3) and Imperator (group 6). Assignment accuracy on the cultivar level was 71% for group 6 and 100% for group 1 and group 3. Remaining groups were more heterogeneous with regard to classification of specific market types. For *DcS-*ILPs group 4 was the most numerous (Additional file 9: Table S8). Within this group Danvers, Paris Market and Guerande types prevailed. It comprised six cultivars of the Danvers type, four of the Paris Market type, four of the Guerande type together with two cultivars of the Nantes type NA1 and NA5), one of the Amsterdam type (AM5) and one cultivar of fodder carrot (FO2). The second most numerous group was comprised of seven cultivars of the Autumn King type, four cultivars of the Berlikum type and one cultivar of the fodder carrot (FO4). Group 7 was comprised of seven cultivars of the Nantes type. Group 8 comprised cultivars of the St. Valery type together with one Long Orange cultivar (LO1), three cultivars of the Autumn King type (AU1, AU7 and FL1), one cultivar of Berlikum type (BE5), one cultivar of Chantenay type (CH9) and one cultivar of fodder carrot (FO4). The least numerous group comprised only three cultivars, two of which were of the Paris Market type (PA1, PA8) and one of the Danvers type (DA6).

For SNPs group 2 was the most numerous and comprised 19 cultivars of the Autumn King and Berlikum types together with fodder carrot (Additional file 10: Table S9). Thirteen cultivars of the Paris Market and Guerande types and one cultivar of the Chantenay type (CH9) were placed in group 5, whereas 8 cultivars of the Danvers type together with four cultivars, each of different type (BE5, CH6, NA8, FO2), were placed in group 4. Seven cultivars belonging to the Nantes type were grouped together with one cultivar of the Amsterdam type (AM5). Group 8 comprised only four cultivars: two of them of the St. Valery type, one of the Nantes type (NA6) and one of the Long Orange type (LO1).

## Discussion

Previous studies carried out on diverse collections of wild and cultivated carrots suggested there was no distinctive genetic structure within neither wild nor cultivated (western and eastern) carrot [3, 8, 9, 11, 13, 16, 20]. Nonetheless, the above-mentioned studies were carried out on large sets of very diverse carrot germplasm. Because of the apparent and very distinctive genetic differences between wild and cultivated gene pools, they might not have been able to detect the structure of genetic diversity present within the group of western OP cultivars. Historically, the first of the market classes of the western cultivated carrot were selected in the eighteenth century from groups of cultivars showing similar storage root morphologies [21]. As such, the cultivars classified to one market class might have been of relatively close kin. However, as previously reported by Iorizzo et al. [1], no significant bottleneck was observed in the cultivated carrot, pointing at a possible continuous gene flow between the wild and the cultivated pools, and very likely also among different cultivars. Conventional selection based on the plant morphology, leading to the development of the existing types of carrot cultivars showing distinct morphological and agronomic characteristics, apparently allowed retention of significant amounts of genetic heterogeneity within OP cultivars.

The codominant *DcS*-ILP marker system exploited in the present study might reveal genetic variability which arose more recently as *DcSto* MITEs show extreme insertional polymorphism within the carrot genome [19, 22]. Possibly, their recent mobilisation could have led to the genetic diversification within the western carrot gene pool. Despite many advantages, high throughput molecular marker systems, such as SNPs or DArTs are not able to detect transposable element (TE) insertion-derived variability. The resolving power of the *DcS*-ILP panel was previously demonstrated on the collection of 23 OP cultivars of western type carrot [18]. The panel of high quality SNPs bears advantages of cost-efficient throughput sequencing-derived markers but is reduced to ca. 2300 loci evenly-distributed across the genome and referred to the high-quality genome assembly [1], thus providing time- and computing efficiency when exploited for the evaluation of genetic structure within the larger datasets. Both panels of markers can easily be extended by additional loci to gain extra biological information or to possibly modify the resolution of population structure.

The results of the AMOVA, together with high values of H_O_, on both cultivar and market class level indicated a significantly higher level of intra-cultivar genetic diversity, mainly contributing to the overall genetic diversity observed in the investigated collection of cultivars. This observation is in accordance with previous studies of Maksylewicz and Baranski [10], as they indicated that almost two third of the of total variation observed in highly diverse collection of carrot cultivars and landraces was attributed to intra-population variation. The values of inbreeding coefficients in the collections of both advanced cultivars and landraces in the studies carried out by Baranski et al. [9] and Maksylewicz and Baranski [10] indicated the excess of homozygous loci that could suggest the repeated selfing during breeding programs aimed at the production of uniform, advanced cultivars. However, using a much more robust set of polymorphisms we did not observe positive inbreeding coefficients in our collection of OP cultivars.

**Fig. 8:**
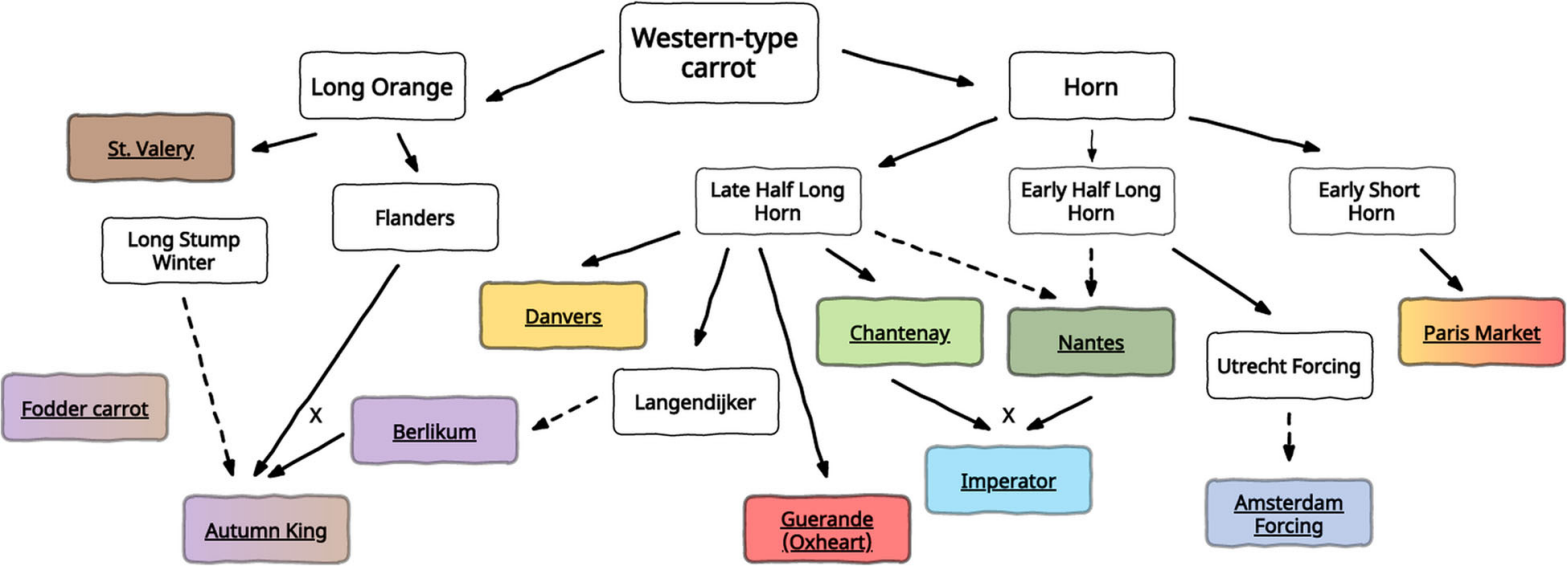
Schematic representation of the origin of main types of Western carrot as proposed by Banga [14]. Solid arrows show direction of the development of new cultivar types. Punctuated arrows indicate possible origin of the particular type. Underlined names indicate the types of cultivars investigated in the present study. Colours of the boxes represent particular market types or groups of market types clustered using DAPC method for both *DcS*-ILP and SNP data (see Fig. 7)

Cultivars classified as Chantenay, Amsterdam and Paris Market types showed lower gene diversity (measured as H_E_) among the 11 predefined market classes, whereas cultivars classified to the Berlikum, St. Valery and Imperator types were among the most heterogenous. STRUCTURE clustering led to the decrease in the most probable number of groups from 11 predefined market classes to the most probable three to five or seven clusters. Non-model DAPC grouping also indicated the lesser number of relatively homogenous groups in the examined collection of cultivars. The choice of the most probable number of clusters was more ambiguous for *DcS*-ILP markers as the differences in the ΔK value were relatively small, but generally the increase of the number of defined clusters resulted in lower fraction of the unassigned cultivars together with the increase of the average membership coefficient (Q) within the clusters. This tendency was reversed in the case of SNP genotyping data. The more clusters were extracted, the lower mean values of Q within the groups were observed. Nonetheless, most of the cultivars belonging to the Amsterdam and Chantenay types were always clearly separated from other varieties and characterized by the highest values of within-group Q together with the lowest within-group H_E_. According to the classification of carrot market types proposed by Banga [14] both the Amsterdam and the Chantenay types represent the ‘Horn’ group that comprises a vast selection of high-quality carrot cultivars subdivided to at least eight market classes (Fig. 8). The Amsterdam market type refers to forcing carrot cultivars grown under covers or for early production in the open. The use of Amsterdam cultivars was originally limited to an early production under covers. Breeders were seeking plant material characterised by a high yield and considerable length, together with the vigour being the key feature in forcing varieties. In the nineteenth century only few varieties could meet the expectations, with *Utrecht Forcing* among them. The modern Amsterdam OP cultivars are believed to be direct descendants of the *Utrecht Forcing* [14].

To date, there is no evidence pointing out that any other breeding material was used to develop Amsterdam cultivars. It is in accordance with our results of STRUCTURE and DAPC clustering, indicating a relatively strong distinctiveness of Amsterdam carrots from other market types, possibly allowing preservation of its specific agricultural characteristics. The admixed nature of *Amsterdamska* cv. possibly reflects the use of Nantes breeding material in the course of the cultivar development. The Chantenay market type comprises cultivars developed for the production between half-summer and late winter carrots. This type is considered as a deviation from main types representing ‘Late Half Long Horn’ group of market types and was developed as a parallel selection to Guerande type and are believed to originate from *la race de Hollande* cv [14].. However, the majority of Chantenay cultivars was grouped in one clearly distinctive cluster with very high membership values. Regardless of the molecular marker genotyping system, two cultivars, i.e. *Chantenay Long Type* and *Criolla*, were characterized by high levels of admixture. Interestingly, in both cultivars the major genetic components were derived from the market classes with shared ancestor according to the classification proposed by Banga [14]. The two major genetic components of *Chantenay Long Type* cultivar pointed towards the cross between breeding materials belonging to the Danvers and Imperator types. Since *Chantenay Long Type* originated in the U.S., the breeding material of Danvers type could be introduced in the course of the cultivar development, especially because this market type originated in the U.S. in 1870s, and is still used for bunching [14]. The relatively high proportion of genetic components originating from Imperator might be the effect of the crosses between Chantenay and Imperator aimed at obtaining longer storage root. Similarly, the reason for clustering of *Criolla* cultivar (originally classified as Chantenay) with cultivars of the Danvers type could be in the use of the most easily accessible parental breeding material originating from North America. It highlights possible discrepancies between the passport data attributing a cultivar to a particular market class relative to its phenotype and its actual pedigree. Ma et al. [13] reported that Chinese orange carrots, sharing many morphological characteristics with western orange carrots, clustered with Chinese red carrots, suggesting that Chinese orange cultivars and landraces could have emerged from original Asian carrot varieties. It shows that similar phenotypic traits can result from selection from different gene pools. Thus, the presented results possibly reveal the actual genetic diversity within the western carrot gene pool, coupled with remarkable intra-cultivar heterogeneity and significant levels of admixture.

## Conclusions

The aim of the current study was to detect possible genetic structure underlying phenotypic differences among market types of western carrot. We exploited two molecular marker systems, SNPs and TE insertion-derived DcS-ILPs, to provide the tool for time- and cost-efficient evaluation of larger datasets. Both marker systems enabled detection of substantial variation among carrot plants of different market types, therefore can be used in germplasm characterization and analysis of genome relationships. Both model-based STRUCTURE clustering and non-model DAPC grouping indicated the reduction in the number of relatively homogenous groups of OP cultivars in comparison with the classification based primarily on phenotypic traits. The presented results likely reveal the actual genetic diversity within the western carrot gene pool and point at possible discrepancies within the cultivars’ passport data.

## Methods

### Plant material and DNA extraction

Carrot open-pollinated cultivars used in this study were obtained from the Warwick Genetic Resource Unit (WGRU) (Additional file 11**: Table S10**). A total of 390 plants representing 78 OP western-type carrot cultivars (five plants per cultivar) of various tap root shape and market types were grown in the field in Gołębiew (Poland), in 2014 and 2016, under standard agricultural practice, optimally irrigated, fertilized and protected from pathogens. DNA was isolated from fresh young leaves using a modified CTAB protocol (Briard et al., 2000).

### *DcS-*ILP genotyping and SNP discovery by GBS

Genotyping with the use of 93 *DcS*-ILP markers (Additional file 12: Table S11) was performed as described by Stelmach et al. [18]. The *DcS*- ILP marker profiles were scored manually. Each allele was scored as: 1 (empty insertion site), 2 (occupied insertion site) or 0 (lack of amplification). The codominant marker matrix with diploid individuals was created (Additional file 13: Table S12). For SNP discovery plants were genotyped using genotyping-by-sequencing (GBS) at the University of Wisconsin-Madison Biotech Center, carried out as described by Ellison et al. [23]. SNPs were called using Tassel 5.2.31 [24, 25] and the reference carrot genome LNRQ00000000.1 [1]. Polymorphisms were filtered using the VCFtools [26]. Only high quality SNPs (parameters: --max-alleles 2 --max-missing-count 95 --maf 0.1 --minDP 8) were retained and the resulting vcf file was subsequently recoded to the STRUCTURE format matrix using plink 1.9 software [27] (Additional file 14: Table S1).

### Data analysis

Genetic diversity indices such as: number of alleles (N_a_), number of effective alleles (N_e_), observed heterozygosity (H_O_) and expected heterozygosity (H_E_) were calculated for both *DcS*-ILP and SNP codominant marker systems using GenAlEx 6.51 [28]. Pairwise F_ST_ was estimated using FinePop2 R package [29]. Genetic diversity structure was investigated with STRUCTURE 2.3 [30]. Bayesian clustering was carried out on both *DcS*-ILP and SNP genotyping data matrices. The length of the burn-in period was set to 100,000 and the number of Markov Chain Monte Carlo (MCMC) replications after the burn-in were assigned at 500,000 for each number of clusters (K) set from 2 to 11 (the number of the predefined market classes). Five independent iterations with an admixture and correlated allele frequencies model were performed for each simulated value of *K. no* prior knowledge about the origin of the analyzed populations was used. The most informative K was identified using ΔK value as described by Evanno et al. [31]. To evaluate differentiation among the most probable number of subpopulations, Nei’s genetic distances and pairwise F_ST_ estimations were calculated in GenAlEx 6.51. Analysis of molecular variance (AMOVA) was also carried out on codominant genotyping distance matrices in GenAlEx 6.51 with 999 permutations. To provide an assessment of genetic diversity of the studied collection without prior assumptions on the population structure, we conducted Principal Component Analysis (PCA) followed by Discriminant Analysis of Principal Components (DAPC) using *adegenet* 2.1.1 R package [32]. Analyses were carried out on both *DcS*-ILP and SNP datasets. In DAPC, the optimal numbers of retained principal components were determined using a cross-validation (*xval* function implemented in *adegenet*). 150 and 60 principal components explaining 76% (SNP) and 89% (*DcS*-ILP) of the total variance were retained for the DAPC analysis of SNP and *DcS*-ILP datasets, respectively. The number of groups was determined de novo using *find.clusters()* function implemented in *adegenet*. The optimal K was selected based on the decreasing values of Bayesian Information Criterion (BIC). Individuals were then assigned to the clusters.

## Supplementary Information

**Additional file 1: Figure S1.** Genomic distribution of SNP and *DcS*-ILP markers on nine chromosomes of the carrot genome; Legend: The black vertical bars correspond to the position of SNP and DcS-ILP markers; the names of *DcS*-ILPs are bold maroon.

**Additional file 2: Table S1.** *DcS*-ILP panel summary statistics for the analysed collection of carrot cultivars.

**Additional file 3: Table S2.** SNP panel summary statistics for the analysed collection of carrot cultivars.

**Additional file 4: Table S3.** Comparison of the percentage of polymorphic loci observed within analysed cultivars.

**Additional file 5: Table S4.** Mean H_O_ and H_E_ estimates and pairwise F_ST_ values obtained for the predefined market classes.

**Additional file 6: Table S5.** Pairwise cultivar estimates of F_ST_ based on 93 *DcS*-ILP markers.

**Additional file 7: Table S6.** Pairwise cultivar estimates of F_ST_ based on 2354 SNP markers.

**Additional file 8: Table S7.** Estimation of the optimum number of clusters based on Evanno’s ∆K method.

**Additional file 9: Table S8.** Results of DAPC grouping carried out on *DcS*-ILP genotyping data.

**Additional file 10: Table S9.** Results of DAPC grouping carried out on SNP genotyping data.

**Additional file 11: Table S10.** Characteristics of the 78 carrot cultivars used in the study.

**Additional file 12: Table S11.** Description of 93 *DcS*-ILP markers used for genotyping the collection of 78 OP carrot cultivars.

**Additional file 13: Table S12.** The codominant *DcS*-ILP marker matrix obtained for the collection of 390 carrot plants.

**Additional file 14: Table S13.** The codominant SNP marker matrix obtained for the collection of 390 carrot plants.

## Data Availability

The datasets supporting the conclusions of this article are included within the article and its additional files or are available from the corresponding author on reasonable request.
