# Supplementary figures and images for: Genetic diversity structure of western-type carrots
Source: BMC Plant Biol. 2021 Apr 26;21:200. doi: 10.1186/s12870-021-02980-0 (PMC8077844; doi:10.1186/s12870-021-02980-0)

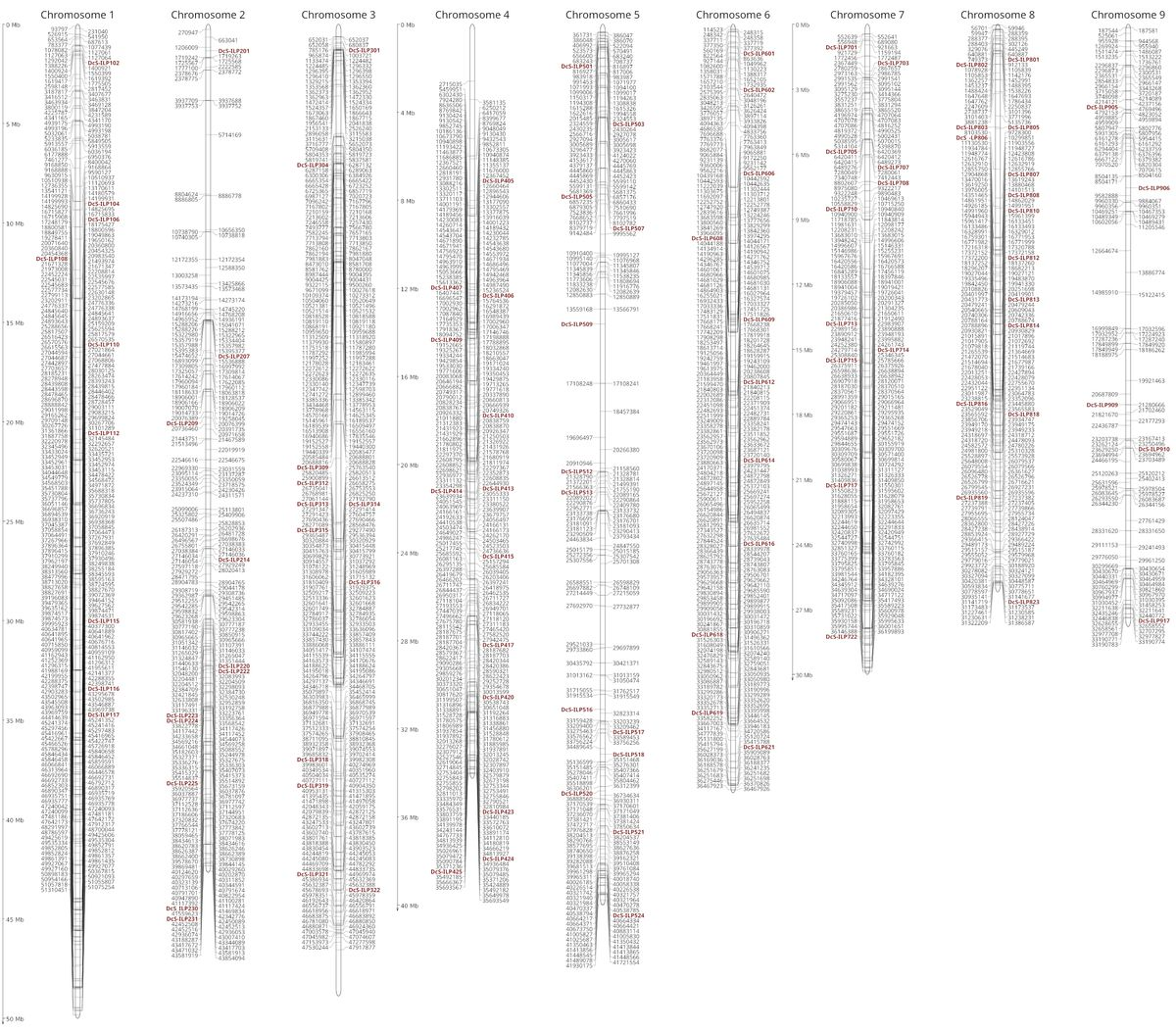

Supplement: Supplementary file 1 — Additional file 1: Figure S1. Genomic distribution of SNP and DcS-ILP markers on nine chromosomes of the carrot genome; Legend: The black vertical bars correspond to the position of SNP and DcS-ILP markers; the names of DcS-ILPs are bold maroon. [file 12870_2021_2980_MOESM1_ESM.tif]
